# The association between dose of hemodialysis and patients mortality in a prospective cohort study

**DOI:** 10.1038/s41598-022-17943-0

**Published:** 2022-08-12

**Authors:** Shu-Xin Liu, Zhi-Hong Wang, Shuang Zhang, Jia Xiao, Lian-Lian You, Yu Zhang, Cui Dong, Xue-Na Wang, Zhen-Zhen Wang, Sheng-Nan Wang, Jia-Ni Song, Xiu-Nan Zhao, Xin-Yi Yan, Shu-Fan Yu, Yi-Nan Zhang

**Affiliations:** 1 Department of Nephrology, Dalian Municipal Central Hospital, No.826, Xinan Road, Dalian, Liaoning 116033 China; 2 Dalian Key Laboratory of Intelligent Blood Purification, Dalian Municipal Central Hospital, No.826, Xinan Road, Dalian, Liaoning 116033, China

**Keywords:** Medical research, Nephrology, Urology

## Abstract

Dialysis adequacy is a known risk factor for mortality in maintenance hemodialysis (MHD) patients. However, the optimal dialysis dose remains controversial. Therefore, we aimed to explore the relationship between dialysis dose and all-cause and cardiovascular disease (CVD) mortality among MHD. We examined the associations of dialysis dose with mortality in a cohort (n = 558) of MHD patients from 31 December 2015 to 31 December 2020. Dialysis adequacy was assessed using baseline Single-pool Kt/V_urea_ (spKt/V), which was categorized into three groups, and the lowest dose group was used as the reference category. Hazard ratios (HRs) and 95% confidence intervals (CIs) were calculated using Cox proportional hazards regression models. A total of 214 patients died (64.5% for CVD). Compared with the low-dose group, high-dose group could reduce the risk of all-cause mortality by 33% (HR = 0.67, 95% CI: 0.47–0.98). Of note, when stratification by age, high-dose group was associated with both lower all-cause (HR = 0.46, 95% CI: 0.26–0.81) and CVD mortality (HR = 0.42, 95% CI: 0.20–0.88) among patients with age below 65 years. When stratification by dialysis age, high-dose group was associated with decreased risk of CVD mortality (HR = 0.43, 95% CI: 0.20–0.91) among patients with dialysis age over 60 months. spKt/V is a simple index of hemodialysis dose used in clinical practice and a useful modifiable factor in predicting the risk of death, especially in MHD patients under 65 years old or dialysis age more than 60 months.

## Introduction

Chronic kidney disease (CKD) is a global public health problem, and one of the major adverse outcomes is entering end-stage renal disease (ESRD). It had been revealed that the prevalence of ESRD was estimated as 0.07% (95% UI 0.06 to 0.08) in the world's population^1^, and the prevalence of hemodialysis (HD) patients in China was 379.1 per million population in 2017^2^. The prevalence of ESRD continues to increase, besides, its prognosis is poor. In 2018, the adjusted mortality rate for MHD patients in the United States was 164.6 per thousand patient-years, and cardiovascular disease (CVD) was the most common cause of death, accounting for 42.2%^3^.

There are many risk factors affecting the survival of MHD patients, among which dialysis adequacy is one of the most important and actionable laboratory variables^4,5^. Dialysis adequacy measured by single-pool Kt/V_urea_ (spKt/V), which is urea clearance multiplied by duration of treatment session and normalized for urea distribution volume, is the most frequently applied measure of delivered dialysis dose^6^. The clinical practice guidelines in the United States, Europe, Japan and Canada recommend a target Kt/V dose of 1.2–1.4 for patients dialyzing thrice weekly^7–10^.

However, the relationship between the delivery dose of hemodialysis and patient mortality remains controversial. Several studies indicated that lower than the recommended Kt/V may increase mortality^11–15^. In a 5-year cohort study of more than 88,000 MHD patients in the United States conducted by Jessica E Miller et al., it had been reported that the greatest survival gain of higher HD dose was associated with a Kt/V approaching the 1.6 to 1.8 range^16^. In contrast, several studies had shown that increasing dialysis dose has no effect on survival gain and no predictive effect on cardiovascular death^17–19^. The hemodialysis (HEMO) study compared the outcomes of patients at high dialysis doses (target spKt/V approximately 1.65) with those at standard dialysis doses (target spKt/V approximately 1.25) and showed no significant differences in all-cause and cardiovascular deaths^20^. These inconsistencies may be attributed to the influence of different races, duration of follow-up, different nutritional status and other factors on Kt/V. Therefore, we hypothesis that higher dose of hemodialysis might lower the risk of death.

Up to now, few studies have examined the effects of different doses of spKt/V on all-cause death and cardiovascular death among MHD patients in China. Therefore, we conducted this long-term follow-up prospective cohort study to determine whether different dialysis dose could influence all-cause mortality and CVD mortality among MHD patients in a large hemodialysis center.

## Materials and methods

### Study population

A double-blind prospective cohort study was conducted at the Blood Purification Center of Dalian Municipal Central Hospital, which is the largest hemodialysis center of the three provinces in northeast China. The inclusion criteria were hemodialysis patients aged ≥ 18 years, receiving stable hemodialysis for ≥ 12 weeks. Patients with acute infection, malignancy, or cardiovascular event in the previous 3 months were excluded. Patients dropped out or who were switched over to renal transplantation were also excluded. All patients underwent three hemodialysis sessions per week and each session was 4 h. The dialysate flow was 500 mL/min. The blood flow rate was between180 and 300 mL/min. Demographic and clinical characteristics in 2016 were collected. The observation study was conducted until 31 December 2020. All subjects enrolled in this research have given their informed consent which has been approved by the research ethics committee of Dalian Municipal Central Hospital.

### Laboratory measurements

Blood samples were drawn during the midweek dialysis day. Venous blood samples for routine laboratory determinations were obtained immediately before the dialysis treatment and shipped to the laboratory for analysis. The post-dialysis samples was also collected. To avoid dilution to obtain the post-dialysis urea, the ultrafiltration rate was set to zero and blood pump rate was reduced to 100 ml/min. Fifteen seconds after reducing the blood flow, the sample was then drawn from the arterial needle tubing^21^. Hemoglobin was measured using sodium dodecyl lauryl sulfate. Other laboratory values were measured using an ADVIA 2400 Automatic Biochemistry Analyzer (Siemens, Germany). Laboratory parameters were measured quarterly, including hemoglobin, alanine aminotransferase, albumin, alkaline phosphatase, urea nitrogen, creatinine, potassium, sodium, chlorine, phosphorus, calcium, total cholesterols, and triglyceride. The baseline for each patient was the average calendar quarter of 3 times.

Dialysis adequacy was determined using the spKt/V which was calculated quarterly using the second generation equation of Daugirdas^22^, from pre- and post-hemodialysis blood urea, as follows: Kt/V = − ln(R − 0.008 × t) + [(4 − 3.5 × R) × UF/W], where R is the ratio of post-dialysis to pre-dialysis serum urea nitrogen concentration; t is duration of hemodialysis (in hours); UF is the amount of ultrafiltration (in liters) during the hemodialysis session; and W is the post-dialysis weight (in kilograms).

### Outcome evaluation

For the duration of the study, the primary outcome for our analysis was all-cause mortality. The secondary outcome was cardiovascular mortality. All deaths were accurately recorded on TSS version 2.0 (Therapy Support Suite, Baden Humboldt, German), hospital records on BS-EAP (version 5.5; B-soft Enterprise Application Portal, Hang Zhou, China) and CNRDS (version 2017; Chinese National Renal Data System, Beijing, China). Cardiovascular mortality included deaths due to coronary events, sudden cardiac death, heart failure, myocardial ischemia, arrhythmias, and cerebrovascular accidents. Each patient were followed from entry until death or the earliest of the following censoring events: kidney transplantation, withdrew from the study, or the end of follow up.

### Statistical analysis

spKt/V was categorized into three groups (the low-dose group: < 1.2, the middle-dose group: 1.2–1.4; the high-dose group: > 1.4)^23^, and the low-dose group was used as the reference category. The normality of all continuous variables was evaluated using the Shapiro–Wilk statistic. The results of continuous variables are expressed as the mean ± standard deviation (SD) or median (quartile1, quartile3), and intergroup comparisons were analyzed using one-way ANOVA for normally distributed data or the Kruskal–Wallis H tests for non-normally distributed data. Categorical variables are expressed as the count with percentage, and differences between the two groups were examined using chi-square tests.

Survival curves were calculated by the Kaplan–Meier method, and differences between the curves were analyzed using the log-rank test. We carried out crude and multivariable Cox proportional hazard regression models to calculate hazards ratios (HRs) and the corresponding 95% confidence intervals (CIs). We used the Schoenfeld residual test to verify the assumption of proportional hazards in the Cox analysis, and no violations were found (all *P* > 0.05). The models were without any adjustment (model 1); adjusted for age, gender and duration of dialysis (model 2); and additionally adjusted for heart failure, hemoglobin, albumin, creatinine, total cholesterol and primary disease on the basis of model 2 (model 3). In addition, we did subgroup analysis to assess potential effect modification by age (< 65 and ≥ 65 years) and duration of dialysis (< 36 months, 36–60 months and ≥ 60 months). All analyses were carried out using SAS software, version 9.4 (SAS Institute, Inc., Cary, NC, USA). Statistical significance was set at a *P* < 0.05 and based on a two-sided test.

### Ethics approval and consent to participate

The study protocol was approved by the institutional medical ethics committee of the Dalian Municipal Central Hospital. All participants or a next of kin of the participants were provided written informed consent before data collection. The present study was performed in accordance with the Declaration of Helsinki.

## Results

We excluded patients without spKt/V value and other variables (n = 77) and age below18 years (n = 1). A total of 558 patients on MHD were enrolled, with a median age of 58(47–68) years and 55.6% were males. The median follow-up duration was 61(34–61) months, during this period, 214 patients died [138(64.5%) cardiac]. The median dialysis age was 47 (18–78) months. The primary disease was 37.3% glomerulonephritis, 26.3% diabetic nephropathy, 19.2% hypertensive benign renal arteriosclerosis and 6.8% polycystic kidney, respectively.

The baseline characteristics of three groups are shown in Table 1. The patients were categorized into three groups according to baseline spKt/V: low dose group (spKt/V < 1.2); medium-dose group (1.2 ≤ spKt/V ≤ 1.4); and high-dose group (spKt/V > 1.4). There were differences in gender, primary disease, time on dialysis, hemoglobin, albumin, creatinine, potassium, phosphorus, calcium, total cholesterols, heart failure rate among three groups (*P* < 0.05).

**Table 1:** Baseline characteristics of the study patients.

Characteristic	Study population (n = 558)	Average spKt/V range			
		< 1.2 (n = 155)	1.2–1.4 (n = 182)	> 1.4 (n = 221)	*P*
Age, years	58 (47–68)	58 (46–67)	58 (45–65)	58 (48–70)	0.53
Male, n (%)	310 (55.6)	126 (81.3)	110 (60.4)	74 (33.5)	< 0.01
Time on dialysis, months	47 (18–78)	33 (13–68)	38 (15–71)	63 (32–90)	< 0.01
**Primary disease, n (%)**					< 0.01
Diabetic nephropathy	147 (26.3)	51 (32.9)	50 (27.5)	46 (20.8)	
Glomerulonephritis	208 (37.3)	43 (27.7)	65 (35.7)	100 (45.3)	
Hypertensive benign renal arteriosclerosis	107 (19.2)	35 (22.6)	36 (19.8)	36 (16.3)	
Polycystic kidney	38(6.8)	12 (7.7)	17 (9.3)	9 (4.1)	
Others	58 (10.4)	14 (9.0)	14 (7.7)	30 (13.6)	
Hemoglobin, g/L	107 (98–114)	104 (95.3–113.3)	108 (99–116)	107 (99–113.3)	0.03
Alanine aminotransferase, U/L	10 (7.7–13)	10 (8–13.3)	9.7 (7.5–13.5)	9.3 (7.7–12.7)	0.57
Albumin, g/L	40.2 (38.4–41.8)	40.3 (38.3–42.0)	40.4 (38.9–42.3)	39.8 (38–41.2)	0.02
Alkaline phosphatase, U/L	93.7 (72.7–131)	92.3 (71.3–125.7)	91.8 (72–125.3)	99.3 (74.3–134)	0.17
Urea nitrogen, mmol/L	25 ± 5	24.3 ± 5.8	25.3 ± 4.8	25.2 ± 4.6	0.24
Creatinine, umol/L	935.8 (763.5–1097)	970 (743–1167.3)	986.8 (804–1147)	881 (747–1018.3)	< 0.01
Potassium, mmol/L	5.0 (4.6–5.5)	4.8 (4.5–5.3)	5.1(4.6–5.6)	5 (4.6–5.5)	< 0.01
Sodium, mmol/L	137.1 (135.5–138.9)	137.5 (135.6–139.1)	137.1 (135.6–139)	136.8 (135.2–138.6)	0.18
Chlorine, mmol/L	97.8 (95.8–99.8)	98.1 (96–100)	97.7 (95.6–99.7)	97.7 (96.1–99.7)	0.43
Phosphorus, mmol/L	2.0 (1.7–2.3)	2.1 (1.7–2.4)	2.0 (1.7–2.3)	1.9 (1.7–2.2)	< 0.01
Calcium, mmol/L	2.4 (2.3–2.5)	2.3 (2.3–2.4)	2.4 (2.3–2.5)	2.4 (2.3–2.5)	< 0.01
Total cholesterols, mmol/L	4.2 (3.6–4.8)	4 (3.3–4.5)	4.2 (3.6–4.8)	4.3 (3.8–4.9)	< 0.01
Triglyceride, mmol/L	1.7 (1.2–2.6)	1.9 (1.2–2.9)	1.9 (1.2–2.7)	1.6 (1.2–2.4)	0.12
Ultrafiltration rate, mL/kg/h	2.1 (1.6–2.7)	2.2 (1.5–2.7)	2.1 (1.6–2.7)	2 (1.6–2.5)	0.91
Heart failure	166 (29.8)	53 (34.2)	65 (35.7)	48 (21.8)	< 0.01
Stroke	63 (11.3)	18 (11.6)	20 (11.0)	25 (11.4)	0.98
Tumor	28 (5.0)	9 (5.8)	8 (4.4)	11 (5.0)	0.83

Data are displayed as mean ± standard deviation or median [quartile1 − quartile3] for continuous variables and number (percent) for categorical variables.

*P* values were determined with one-way ANOVA or Kruskal–Wallis H tests for continuous variables and chi-square test for categorical variables.

All statistical tests are two sided.

**Figure 1:**
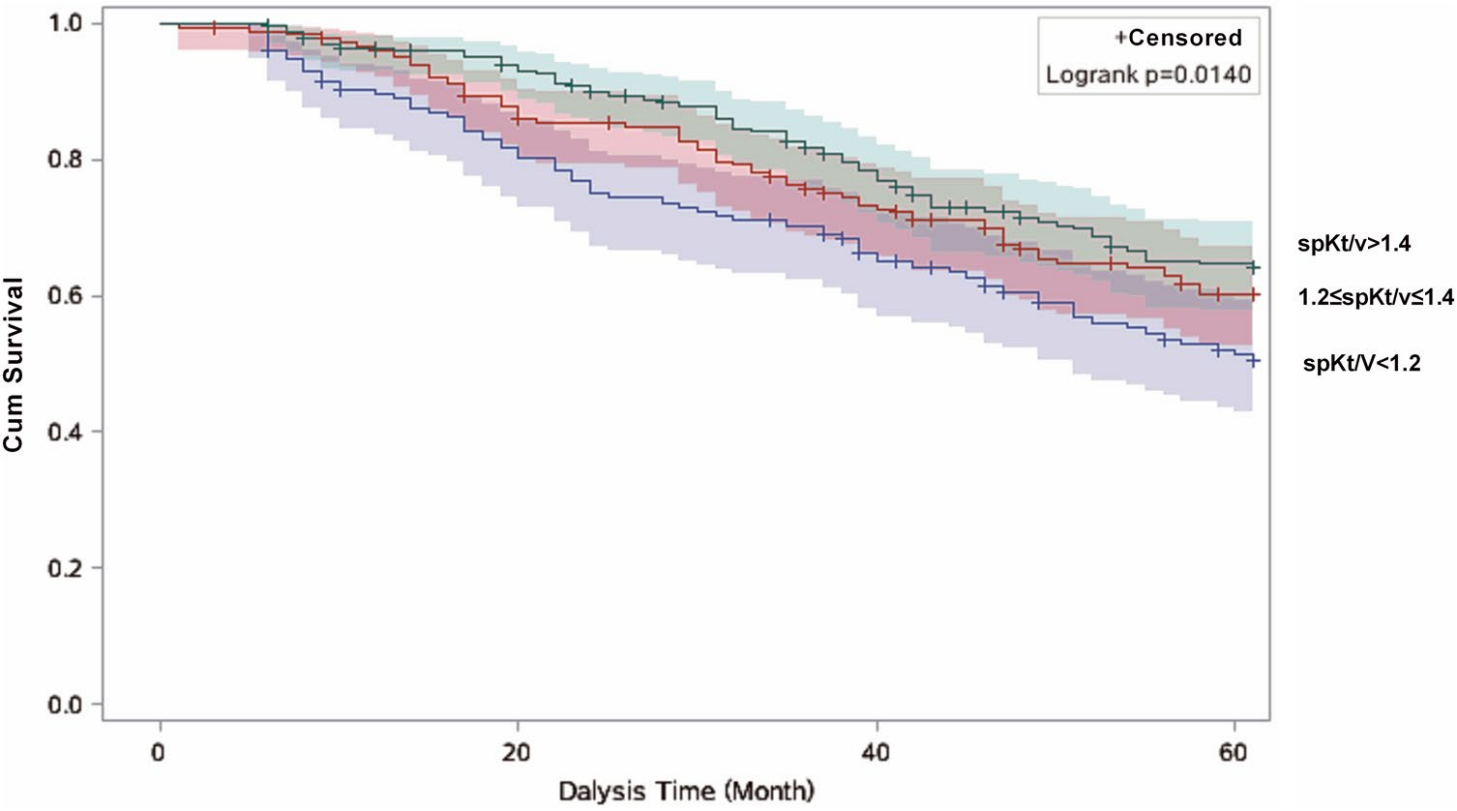
Kaplan–Meier survival estimates of all-cause mortality between the three the dose of hemodialysis subgroups divided by the optimal cutoff value.

**Figure 2:**
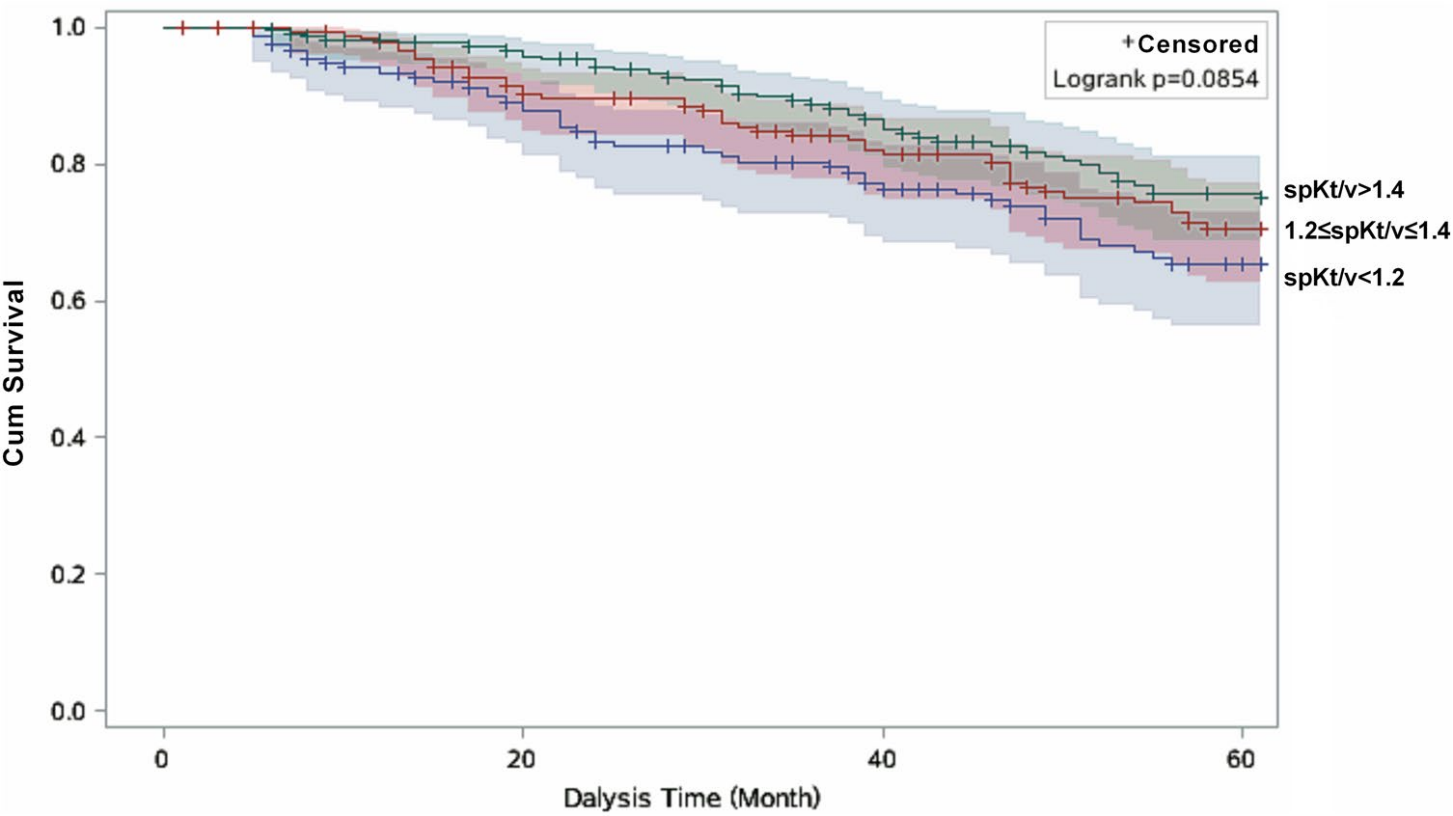
Kaplan–Meier survival estimates of cardiovascular disease mortality between the three the dose of hemodialysis subgroups divided by the optimal cutoff value.

For the total study cohort, Kaplan–Meier curves (Fig. 1) showed that the risk of all-cause death with baseline spKt/V was significantly different among three groups during the follow-up period (log-rank, P = 0.014). However, there was no significant difference between spKt/V and cardiovascular mortality (log-rank, P = 0.085) (Fig. 2). The results of Cox regression analyses were presented in Table 2. Overall, in the fully adjusted model, MHD patients in high-dose group had a reduced risk (HR = 0.67, 95% CI: 0.47–0.98) of all-cause mortality compared to patients in low-does group. However, baseline spKt/V was not significantly related to cardiovascular mortality (HR = 0.68, 95% CI: 0.43–1.09).

**Table 2:** Hazard ratios (95% CI) for the association between dialysis dose and all-cause and cardiovascular disease mortality.

Categories	Model 1^a^ HR (95% CI)	Model 2^b^ HR (95% CI)	Model 3^c^ HR (95% CI)
**All-cause mortality**			
spKt/V < 1.2	1.00 (Ref)	1.00 (Ref)	1.00 (Ref)
1.2 ≤ spKt/V ≤ 1.4	0.73 (0.52–1.02)	0.79 (0.56–1.10)	0.96 (0.68–1.37)
spKt/V > 1.4	0.62 (0.45–0.86)	0.57 (0.40–0.81)	0.67 (0.47–0.98)
**Cardiovascular disease mortality**			
spKt/V < 1.2	1.00 (Ref)	1.00 (Ref)	1.00 (Ref)
1.2 ≤ spKt/V ≤ 1.4	0.79 (0.52–1.19)	0.86 (0.57–1.30)	1.04 (0.67–1.61)
spKt/V > 1.4	0.63 (0.42–0.95)	0.57 (0.37–0.90)	0.68 (0.43–1.09)

*CI* confidence interval, *HR* hazards ratio, *Ref*, reference.

^a^Model 1: Crude model.

^b^Model 2: Adjusted for age, gender and duration of dialysis.

^c^Model 3: Adjusted for age, gender, duration of dialysis, heart failure, hemoglobin, albumin, creatinine, total cholesterol and primary disease.

We also performed subgroup analyses between dialyses dose and all-cause mortality by age. In the model 3, the hazard ratio decreased with increasing dialysis dose. High dialysis dose shows a significant protective effect on all-cause mortality (HR = 0.46, 95% CI: 0.26–0.81) and CVD mortality (HR = 0.42, 95% CI: 0.20–0.88) in younger age patients (< 65 years). However, the similar result was not observed in older age patients (≥ 65 years) (Table 3).

**Table 3:** Hazard ratios (95% CI) for the association between dialysis dose and all-cause and cardiovascular disease mortality by age.

Categories	Model 1 HR (95% CI)	Model 2 HR (95% CI)	Model 3 HR (95% CI)
**< 65**			
All-cause mortality			
spKt/V < 1.2	1.00 (Ref)	1.00 (Ref)	1.00 (Ref)
1.2 ≤ spKt/V ≤ 1.4	0.81 (0.53–1.24)	0.76 (0.49–1.17)	0.92 (0.58–1.46)
spKt/V > 1.4	0.47 (0.29–0.75)	0.39 (0.23–0.68)	0.46 (0.26–0.81)
Cardiovascular disease mortality			
spKt/V < 1.2	1.00 (Ref)	1.00 (Ref)	1.00 (Ref)
1.2 ≤ spKt/V ≤ 1.4	0.90 (0.53–1.53)	0.84 (0.48–1.45)	1.01 (0.57–1.81)
spKt/V > 1.4	0.44 (0.24–0.82)	0.36 (0.18–0.74)	0.42 (0.20–0.88)
**≥ 65**			
All-cause mortality			
spKt/V < 1.2	1.00 (Ref)	1.00 (Ref)	1.00 (Ref)
1.2 ≤ spKt/V ≤ 1.4	0.65 (0.38–1.10)	0.67 (0.39–1.15)	0.81 (0.46–1.44)
spKt/V > 1.4	0.70 (0.44–1.10)	0.73 (0.45–1.17)	0.84 (0.50–1.40)
Cardiovascular disease mortality			
spKt/V < 1.2	1.00 (Ref)	1.00 (Ref)	1.00 (Ref)
1.2 ≤ spKt/V ≤ 1.4	0.67 (0.35–1.29)	0.70 (0.36–1.35)	0.89 (0.44–1.82)
spKt/V > 1.4	0.72 (0.41–1.27)	0.75 (0.41–1.36)	0.90 (0.47–1.72)

*CI* confidence interval, *HR* hazards ratio, *Ref* reference.

^a^Model 1: Crude model.

^b^Model 2: Adjusted for gender and duration of dialysis.

^c^Model 3: Adjusted for gender, duration of dialysis, heart failure, hemoglobin, albumin, creatinine, total cholesterol and primary disease.

In subgroup stratified by dialysis age (Table 4), compared with the low dose group, the high dose group significantly reduced the risk of CVD mortality by 57% (HR = 0.43, 95% CI: 0.20–0.91) for patients with the dialysis age > 60 months.

**Table 4:** Hazard ratios (95% CI) for the association between dialysis dose and all-cause and cardiovascular disease mortality by duration of dialysis.

Categories	Model 1 HR (95% CI)	Model 2 HR (95% CI)	Model 3 HR (95% CI)
**< 36 (months)**			
All-cause mortality			
spKt/V < 1.2	1.00 (Ref)	1.00 (Ref)	1.00 (Ref)
1.2 ≤ spKt/V ≤ 1.4	0.76 (0.48–1.21)	0.86 (0.54–1.37)	0.93 (0.57–1.51)
spKt/V > 1.4	0.56 (0.32–0.96)	0.61 (0.35–1.07)	0.61 (0.34–1.09)
Cardiovascular disease mortality			
spKt/V < 1.2	1.00 (Ref)	1.00 (Ref)	1.00 (Ref)
1.2 ≤ spKt/V ≤ 1.4	0.93 (0.52–1.66)	1.07 (0.60–1.91)	1.14 (0.61–2.12)
spKt/V > 1.4	0.57 (0.28–1.18)	0.62 (0.29–1.30)	0.64 (0.30–1.37)
**36–60 (months)**			
All-cause mortality			
spKt/V < 1.2	1.00 (Ref)	1.00 (Ref)	1.00 (Ref)
1.2 ≤ spKt/V ≤ 1.4	0.65 (0.26–1.64)	0.40 (0.15–1.06)	0.51 (0.17–1.54)
spKt/V > 1.4	0.62 (0.27–1.43)	0.38 (0.15–0.95)	0.44 (0.15–1.27)
Cardiovascular disease mortality			
spKt/V < 1.2	1.00 (Ref)	1.00 (Ref)	1.00 (Ref)
1.2 ≤ spKt/V ≤ 1.4	0.99 (0.27–3.69)	0.63 (0.16–2.45)	0.87 (0.18–4.17)
spKt/V > 1.4	1.27 (0.40–4.05)	0.84 (0.24–2.97)	1.07 (0.22–5.17)
**> 60 (months)**			
All-cause mortality			
spKt/V < 1.2	1.00 (Ref)	1.00 (Ref)	1.00 (Ref)
1.2 ≤ spKt/V ≤ 1.4	0.71 (0.41–1.26)	0.91 (0.51–1.63)	1.15 (0.61–2.17)
spKt/V > 1.4	0.67 (0.40–1.10)	0.61 (0.34–1.09)	0.67 (0.35–1.28)
Cardiovascular disease mortality			
spKt/V < 1.2	1.00 (Ref)	1.00 (Ref)	1.00 (Ref)
1.2 ≤ spKt/V ≤ 1.4	0.58 (0.30–1.12)	0.71 (0.36–1.39)	0.83 (0.40–1.74)
spKt/V > 1.4	0.49 (0.27–0.88)	0.43 (0.22–0.86)	0.43 (0.20–0.91)

*CI* confidence interval, *HR* hazards ratio, *Ref* reference.

^a^Model 1: Crude model.

^b^Model 2: Adjusted for age and gender.

^c^Model 3: Adjusted for age, gender, heart failure, hemoglobin, albumin, creatinine, total cholesterol and primary disease.

## Discussion

In the current study we evaluated the independent role of spKt/V and other important clinical variables in all-cause and CVD mortality among MHD patients. It showed the higher spKt/V level can reduce the all-cause mortality during the follow-up period while not for the CVD mortality. Moreover, in the patients whose age below 65 years, the higher spKt/V level not only reduce the risk of all-cause mortality but also CVD mortality. Similarly, patients whose dialysis age over 60 months may benefit from higher spKt/V levels which can reduce the risk of CVD mortality.

The effect of the spKt/V on mortality among patients who undergo MHD is somewhat controversial. Low spKt/V has been strongly associated with higher mortality in numerous studies^11,12,24^. A cohort study of 5784 MHD patients in Japan^11^ found that lower Kt/V was associated with elevated mortality, more so among women. An international prospective cohort study reported that for every 0.1 higher Kt/V, the RR of mortality was 2% lower^25^. Similarly, our study found that spKt/V > 1.4 has a protective effect on all-cause deaths in MHD patients. However, in contradiction to the above findings, the HEMO Study reported that increasing dialysis did not improve clinical outcome in MHD patients^26^. This might be due to different race, spKt/V levels and research period. The mean spKt/V of high-dose group in the HEMO study is 1.67, while the standard of our study is more than 1.4, which is far lower than the HEMO study. Due to the different race, Asians often could not achieve extremely high spKt/V. What’s more, the HEMO study, conducted between 1995 and 2000, was 20 years earlier than our study. As time progresses, the improvement of dialysis technology and the improvement of patients' nutritional status may also make differences.

As for CVD mortality, a prospective study from Brazil^27^ found that higher hemodialysis dose was not associated with CVD mortality among MHD patients, which was similar with our results. However, there were several previous studies showed that higher Kt/V can reduce CVD mortality in MHD patients^28,29^. Bloembergen WE et.al. analyzed 2479 MHD patients in America reporting that for each 0.1 higher Kt/V, the death due to cardiac causes was 12% lower^28^.The difference may be due to different races and years of research (1996 versus 2020). Similarly, a prospective randomized controlled study of twice-weekly MHD patients in Shanghai showed that the incidence of CVD in the high-dose group were decreased^29^. The difference may be due to the frequency of dialysis. Compared with three times a week, infrequent HD treatment exposes patients to several risk factors for cardiovascular events, including hypervolemia, and hyperkalemia. The high-dose group can better improve the risk factors of appeal^30^.

Many studies showed increasing spKt/V, which means adequate dialysis, resulted in better survival^31–33^. Inadequate dialysis was associated with poorer nutritional status^34^ and higher degree of inflammation^35^. Serum albumin and hemoglobin levels were significantly improved after increasing the MHD dose^36^. As we all know, nutritional status, especially serum albumin, is strongly related to the mortality in MHD patients^37,38^. And low-dose dialysis may increase the accumulation of uremic toxins, resulting in suppression of red blood cell production and low response to erythropoiesis-stimulating agents^39,40^. In addition, spKt/V was negatively correlated with left ventricular mass index and an independent risk factor for Left ventricular hypertrophy^41^. This may be related to the control of volume overload, anemia and hypertension by increasing spKt/V. And higher spKt/V can better remove low molecular weight uremic toxins, such as asymmetric dimethylarginine, which can cause uremia Cardiomyopathy and endothelial dysfunction^42^.

Our study had several strengths. First, the sample size is large compared to previous studies^27,33,43^, which allowed us to explore the associations between Kt/V levels, and all-cause and CVD mortality in MHD patients with greater accuracy. Second, this study had a relatively long follow-up time (60 months) to prospectively observe the all-cause and CVD mortality in MHD patients. Third, we used the mean value of the three spKt/V measurements in the first year of follow-up as the baseline value of spKt/V, which can more accurately reflect the baseline level of patients and reduce measurement error. Fourth, subgroup analysis of age and duration of dialysis was also carried out. These results have certain guiding significance for clinical work.

However, we truly have several potential limitations in this study. Firstly, due to the limitation of data, we failed to cover all confounding factors which could influence the morbidity from different aspects, such as the cardiovascular biomarkers like N-terminal pro-B type natriuretic peptide, inflammation factors like C-reactive protein, smoke, drink, body composition, dietary factors, residual kidney function and so on. Besides, there may be unmeasured confounding, including time-dependent confounding factors. Secondly, we use the mean value of spKt/V during the first year of follow-up as a factor, which might be result in immortal time bias. However, we found that the spKt/V value did not change with time, which avoided this bias to a certain extent. Thirdly, just as other cohort studies^44,45^, the exclusion of drop-out subjects may be a source of bias due to study design. Finally, the patients we followed up were all from one single center, which made the conclusion less representative. Multicenter studies covering broader confounding factors should be carried out in the future.

## Conclusions

In conclusion, the present cohort study indicated that higher level of spKt/V might could improve the MHD patient's survival rate, particularly in the patients whose age below 65 years or the duration of dialysis more than 60 months. Our findings may aim in developing guidance for dialysis dose in patients on MHD. Future large prospective studies are required to confirm our findings.

## Data Availability

The datasets used and/or analyzed during the current study are available from the corresponding author upon reasonable request.

## Abbreviations

CKD: Chronic kidney disease
CVD: Cardiovascular disease
CIs: Confidence intervals
ESRD: End-stage renal disease
HRs: Hazard ratios
HD: Hemodialysis
IQR: Interquartile range
MHD: Maintenance hemodialysis
SD: Standard deviation
spKt/V: Single-pool Kt/V_urea_
